# Supplementary figures and images for: Stable reference gene selection for Ophiocordyceps sinensis gene expression studies under different developmental stages and light-induced conditions
Source: PLoS One. 2023 Apr 20;18(4):e0284486. doi: 10.1371/journal.pone.0284486 (PMC10118168; doi:10.1371/journal.pone.0284486)

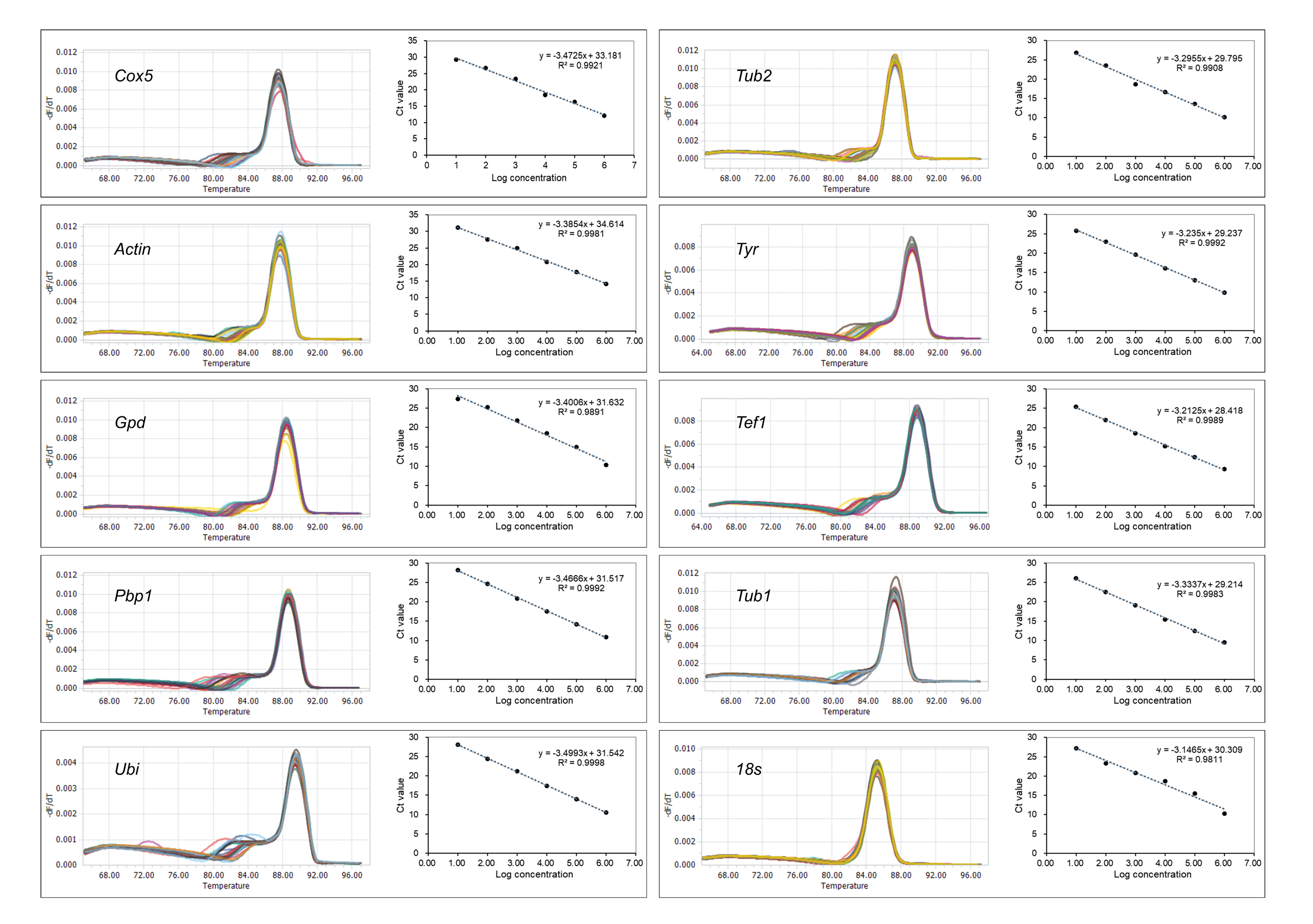

Supplement: S1 Fig — Melting curves were analyzed by LightCycler® 96 Software 1.1 (Roche). The standard curve was amplified from 10-fold diluted standard plasmid. (TIF) [file pone.0284486.s001.tif]
