# Supplementary material for: Stable reference gene selection for Ophiocordyceps sinensis gene expression studies under different developmental stages and light-induced conditions
Source: PLoS One. 2023 Apr 20;18(4):e0284486. doi: 10.1371/journal.pone.0284486 (PMC10118168; doi:10.1371/journal.pone.0284486)
Supplement: Attachment — Submitted filename: Reviewed_ae8f4.docx [file pone.0284486.s002.docx]

# Stable reference gene selection for *Ophiocordyceps* *sinensis* gene expression studies under different developmental stages and light-induced conditions

Chaoqun Tong ^1^, Junhong Wei ^1^, Xianbing Mao ^2^, Guoqing Pan ^1^, Chunfeng Li^1^ * and Zeyang Zhou^1,3^

^1^ State Key Laboratory of Silkworm Genome Biology, Chongqing Key Laboratory of Microsporidia Infection and Prevention, Southwest University, Chongqing 400715, China;

^2^ Chongqing Xinstant Biotechnology Co., Ltd. Chongqing 402231, China;

^3^ College of Life Sciences, Chongqing Normal University, Chongqing 401331, China;

***** Corresponding author

E-mail: [licf@swu.edu.cn](mailto:licf@swu.edu.cn)(C.L.)

# Abstract

The molecular mechanism of Chinese cordyceps formation has received a substantial amount of attention because of its use as a traditional Chinese medicine. Therefore, it is very important to select suitable reference genes to study gene expression. In this study, we selected 10 candidate reference genes and calculated their expression stability using four methods: geNorm, NormFinder, BestKeeper, and Comparative △*C_t_*. After comprehensive analysis of the results of these four methods with RefFinder, we determined that the most stable reference genes during asexual reproduction of *Ophiocordyceps sinensis* were *Tef1* and *Tub*, the most stable reference genes during fruiting body development were *Tyr* and *Cox5*, and the most stable reference genes under light-induced conditions were *Tyr* and *Tef1*. Our study provides stably expressed reference genes that can be used to explore the different proliferation processes and light responses of *O. sinensis*, and represents a foundation for studying the molecular mechanism of Chinese cordyceps formation.

**Keywords:** *Ophiocordyceps sinensis*; reference gene; asexual proliferation; fruiting body development; light induction

# Introduction

Chinese cordyceps, also named ”Dong Chong Xia Cao,” is a traditional Chinese medicine. It is an insect–fungus complex formed by an entomopathogenic fungus, *Ophiocordyceps sinensis*, that parasitizes the larvae of the insect *Thitarodes armoricanus*. Increasing in-depth research has revealed that Chinese cordyceps shows many pharmacological anti-oxidative [1,2], anti-inflammatory [3], hypoglycemic [4], and immunomodulatory [5] actions.

Chinese cordyceps is endemic to the Qinghai–Tibet Plateau and mainly occurs at altitudes between 3000–5000 m [6]. Extreme growth environments limit wild Chinese cordyceps yields, therefore, Chinese researchers have begun to study artificial cultivation of Chinese cordyceps in low altitude areas and have made many breakthroughs [7,8]. In the low-altitude laboratory, the researchers were able to complete the inoculation of *O. sinensis* in the larval stage of the *T. armoricanus*, and induce larval death and mummification to grow fruiting bodies [9]. However, low mummification rates and inconsistent fruiting body development are still major problems that affect artificial cultivation [10]. Consequently, many researchers have focused on fruiting body development [9,11] and the influence of environmental factors (such as temperature and light) on development [12,13].

Through transcriptome and proteome analyses, many differentially expressed genes were identified in different stages of fruiting body development [14], and these genes are involved in MAPK signal transduction, glucose metabolism [15], amino acid metabolism [16], lipid metabolism, and other pathways [17,18]. Simultaneously, it was also found that *O. sinensis* is sensitive to light [19], and the mycelia synthesize melanin under light conditions (especially under UV irradiation) [20]. A large amount of omics data has provided many new targets for *O. sinensis* development. To verify its molecular mechanism, gene expression research is very important and provides an essential foundation.

To study molecular mechanisms, reverse transcription quantitative real-time PCR (RT-qPCR) is a very basic and informative research method. However, some variables may affect the accurate quantification of gene expression [21], such as the differences in materials, RNA extraction and reverse transcription efficiency, and primer design, and a stably expressed reference gene is needed to correct experimental errors [22].

In liquid medium, *O. sinensis* will proliferate asexually in the form of blastospores and hyphae (or mycelial pellets) [14]. During sexual proliferation, *O. sinensis* will first multiply in the hemolymph of the host insect in the form of a hyphal body. The larvae will die and mummify as the hyphal body develops into hyphae; the mycelium preferentially penetrates the larval head to form primordium, and then develops into a fruiting body [23]. During the fruiting body developmental stages, lighting is typically used to control fruiting body length and color. In this study, we collected *O. sinensis* samples at multiple stages of asexual and sexual proliferation, and determined the stability of ten common reference genes using geNorm [21], NormFinder [24], BestKeeper [25], and Comparative △*C_t_* methods [26] to identify the stable reference genes suitable for *O. sinensis* in different research models.

# Materials and Methods

## Strains cultivation and sample collection

For the asexual samples, *O. sinensis* was activated and cultivated with OS1 liquid medium (20% potato infusion, 2% glucose, 1% yeast extract, 0.5% peptone, 0.1% KH_2_PO_4_, 0.025% MgSO_4_), and shaken at 16 °C and 100 rpm for 40 d. The blastospore suspension was collected as seed solution with lens wiping paper, and inoculated in OS1 and 1/4 SDY liquid medium (1% glucose, 0.5% yeast extract, 0.25% peptone), and then shaken at 16 °C and 120 rpm. Light and dark treatments were performed on the samples cultivated with OS1 liquid medium. The blastospore and mycelium samples were harvested in 1/4 SDY liquid medium at 30 d and 45 d, respectively. The loose and dense mycelium balls were harvested in OS1 liquid medium at 30 d and 70 d, respectively.

The larvae infected with *O. sinensis* and the fruiting bodies were reared in a low-altitude laboratory (Jiangjin, Chongqing). The hyphal body samples were collected from the hemolymph of larvae inoculated with *O. sinensis*. Mummified larvae were buried in culture medium for moisture retention fruiting body formation and development. Light (light: dark = 12 h: 12 h) and dark (24 h dark) treatments were performed on the samples after fruiting body formation.

## RNA isolation and cDNA synthesis

The samples were ground in liquid nitrogen, and 200 mg samples were used to isolate total RNA with the Total RNA Kit II (R6934-02，Omega, Norcross, Georgia USA). Then, 2 μg total RNA of each sample was used for reverse transcription synthesis of cDNA with Hifair**®** Ⅲ 1st Strand cDNA Synthesis SuperMix for qPCR (YEASEN, Shanghai, China) and stored at −20 °C after 10-fold dilution. qPCR primers were designed by the NCBI Primer-BLAST tool (https://www.ncbi.nlm.nih.gov/tools/primer-blast/).

## Candidate reference gene selection and primer design

We selected 10 housekeeping genes as candidate reference gene targets, including actin protein (*Actin*), cytochrome c oxidase polypeptide V (*Cox5*), translation elongation factor EF-1 alpha (*Tef1*), polyubiquitin binding protein (*Ubi*), 18s ribosomal (*18s*), glyceraldehyde-3-phosphate dehydrogenase (*Gpd*), polymerase II large subunit (*Rpb1*), tryptophan synthase (*Try*), and tubulin beta chain (*Tub1*, *Tub2*); the tubulin beta chain has two copies in *O. sinensis* that we named *Tub1* and *Tub2*. The candidate genes covered multiple processes such as transcription and translation (*Rpb1* and *Tef1*), protein synthesis and degradation (*18s* and *Ubi*), cytoskeletal structure (*Actin*, *Tub1*, and *Tub2*), and metabolism (*Cox5*, *Gpd*, and *Try*). Details of reference genes and primers are shown in Table 1. Specificity of primer pairs for each candidate gene was confirmed using melting curve analysis (S1 Fig).

**Table 1.** Details of primers for RT-qPCR

| **Gene name** | **Accession number ^1^** | **Primer Sequence (5’-3’) ^2^** | **Amplicon length(bp)** | **Tm**  **(°C)** | **Efficiency (%)** |
| --- | --- | --- | --- | --- | --- |
| *Actin* | KAF4512910.1 | F:TGATGATGCTCCCCGAGCTG | 155 | 64.0 | 97 |
|  |  | R:CACCGTGCTCGATGGGGTAT |  | 62.8 |  |
| *Cox5* | KAF4508291.1 | F:GTCGACCCATGCCATCTCCA | 100 | 63.5 | 94 |
|  |  | R:ACGCAGGGACATCCAAAGGT |  | 61.5 |  |
| *Tef1* | KAF4504225.1 | F:GCGTTGAGGCTTTCACCGAC | 105 | 61.9 | 105 |
|  |  | R:CAGCAGCCTTCTCGACAGAC |  | 59.1 |  |
| *Ubi* | KAF4508947.1 | F:AGATGGCCTCGTTGTCTCGG | 147 | 62.2 | 93 |
|  |  | R:AGAAAAGTGCCGTTGGGGGA |  | 64.0 |  |
| *18s* | KC184161.1 | F:AGATTCAAAGCCAATGCCCC | 129 | 61.4 | 108 |
|  |  | R:TGGCCACTACCCAAACATCG |  | 61.5 |  |
| *Gpd* | KAF4510795.1 | F:TCTTCACCACCACGGACAAG | 143 | 59.7 | 97 |
|  |  | R:GGAAATGACATCGGCCTTGC |  | 61.6 |  |
| *Rpb1* | KAF4510254.1 | F:CAAGCGTGTCGACTTTTCGG | 113 | 61.6 | 94 |
|  |  | R:CAGTCTCGGGATACGTCAGC |  | 59.8 |  |
| *Try* | KAF4507068.1 | F:GAAGGCGCAGATGATGGAGA | 106 | 60.8 | 104 |
|  |  | R:GTCGTTGGCAAAGGGGTAGA |  | 60.0 |  |
| *Tub1* | KAF4510680.1 | F:ATGTCGTCCACCTTTGTCGG | 151 | 60.3 | 100 |
|  |  | R:ACTCGGCTTCGGTAAACTCC |  | 59.6 |  |
| *Tub2* | KAF4504885.1 | F:TATCCAGACAGCCCTTTGCG | 234 | 61.1 | 101 |
|  |  | R:TGGTACTGCTGGTACTCGGA |  | 59.6 |  |

^1^ The accession number comes from NCBI.

^2^ F, forward primer; R, reverse primer.

## RT-qPCR

Quantitative PCR was performed with LightCycler® 96 (Roche, Indianapolis, IN, USA). The PCR mix (10 μL) contained 5 μL Hieff^®^ qPCR SYBR Green Master Mix (YEASEN, Shanghai, China), 0.5 μL per primer, 1 μL 10-fold diluted cDNA, and 3 μL ddH_2_O. The PCR conditions were as follows: 95℃ for 5 min, followed by 40 cycles of 95 °C for 10 s and 60 °C for 30 s. After the final amplification cycle, the melting curve analysis was performed as follows: 65 °C for 60 s, 65–95 °C with 0.5 °C increments, and 97 °C for 10 s. We divided the samples into three groups: asexual proliferation (all liquid fermentation samples), sexual proliferation (samples during fruiting body development), and light treatment (light- and dark-treated samples during liquid fermentation and fruiting body development).

## Statistical analysis

The expression stability of 10 candidate reference genes was analyzed using geNorm [21], NormFinder [24], BestKeeper [25], and Comparative △*C_t_* methods [26]. The overall final ranking was calculated by RefFinder(http://blooge.cn/RefFinder/) [27], which assigns the appropriate weights to individual genes based on the ranking of each program and calculates the geometric mean of their weights for the overall final ranking. The data analysis was performed using LightCycler® 96 Software 1.1 (Roche), and visualization using Origin. Each of the above experiments was independently repeated at least 3 times.

# Results

## Sample collection

Through liquid fermentation, we collected *O. sinensis* samples with five morphologies (Fig 1A): blastospores, hyphae, loose hyphal spheres, and compact hyphal spheres under light and dark conditions. In the early stage of fermentation, 5–10 μm fusiform blastospores proliferated by budding (Fig 1A-a red arrows). After continued cultivation for 45 d, most of the blastospores germinated into hyphae (Fig 1A-b). In OS1 medium, the hyphae aggregated into mycelial pellets (Fig 1A-c). After culturing for 70 days, the mycelial pellets became compact and smooth, which were white under dark conditions (Fig 1A-d) and black under light conditions (Fig 1A-e).

The hyphal body samples were harvested from larval hemolymph (Fig 1B-a) and were fusiform-like blastospores, but the hyphal body length (10–15 μm) was longer than the blastospores (5–10 μm). The mummified larvae were embedded in the culture medium to induce fruiting body formation. Initially, the head shell of the mummified larvae (Fig 1B-b) did not significantly change; however, with proliferation, the fungus preferentially penetrated the larval epidermis at the head shell and form a 'Y-type' hyphal structure (Fig 1B-c). Subsequently, the 'Y-type' hyphal structure swelled and bulged (Fig 1B-d), and finally developed into the primordium (Fig 1B-e). The results of the mummified larvae with primordia exposed to light and dark treatments showed that, in the early stage of fruiting body development, there was no significant difference in fruiting body phenotypes between dark (Fig 1B-f) and light (Fig 1B-g) treatments. When the fruiting body developed to approximately 2 cm, the color of the top of the fruiting body became yellow-brown with light treatment (Fig 1B-i) but was still white under dark treatment. Upon continued cultivation of the fruiting body until it developed to 7 cm, the color of fruiting body was yellow-brown under light treatment (Fig 1B-k), whereas the color only got darker at the top and base under dark treatment (Fig 1B-j). After 40 days of fruiting body development, the middle and upper part of the light-treated fruiting bodies formed bulges (Fig 1B-m) and finally developed into asci (Fig 1B-o), whereas the dark-treated fruiting bodies still had a smooth surface (Fig 1B-i, n).

**Fig 1. Samples of *O. sinensis* under different proliferation conditions.** (**A**) Morphological observation of *O. sinensis* in 1/4 SDY liquid medium, in the early stage of fermentation, fusiform blastospores proliferated by budding (A-a red arrows), after cultivation for 45 d, most of the blastospores germinated into hyphae (A-b). Fermentation culture of O. sinensis in OS1 liquid medium, the hyphae aggregated into mycelial pellets (A-c), after culturing for 70 days, the mycelial pellets became compact and smooth, which were white under dark conditions (A-d) and black under light conditions (A-e). (**B**) Morphological observation of *O. sinensis* at different stages during infecting *T. armoricanus*. (B-a) *O. sinensis* proliferates in *T. armoricanus* larvae hemolymph in the form of hyphal body. The infected larvae died and mummified in 1 month (B-b), and the fungus penetrated the larval epidermis at the head shell and form a 'Y-type' hyphal structure (B-c). The 'Y-type' hyphal structure swelled and bulged (B-d), and finally developed into the primordium (B-e). After primordium formation, mummified larvae were treated in the dark or light environment, respectively. And the samples were taken and photographed at 7 d (dark: Fig 1B-f, light: Fig 1B-g), 18 d (dark: Fig 1B-h, light: Fig 1B-i), 32 d (dark: Fig 1B-j, light: Fig 1B-k), 47 d (dark: Fig 1B-l, light: Fig 1B-m), and 62 d (dark: Fig 1B -n, light: Fig 1B-o) after dark or light treatment, respectively.

## Expression profiling of candidate genes

The expression levels of 10 genes were calculated by quantification cycles and shown with box plots (Fig 2). As introduced in the Materials and Methods section, we divided the samples into three groups (asexual proliferation, sexual proliferation, and light treatment), and the candidate genes had the same expression trend in different groups. Except for the Cq value of *18s* distributed around 10, the expression levels of other genes were in the range of 20–30, which indicated that the expression level of *18s* was significantly higher than that of other genes. In the sexual proliferation and light treatment groups, the Cq value of *Ubi* reached 32.97, which indicated that the expression of *Ubi* was very low in the samples.

**Fig 2.** Candidate gene expression levels under different grouping conditions. (**A**) liquid fermentation samples. (**B**) Sample of fruit body formation process. (**C**) Light or dark treated samples during liquid fermentation or fruiting body formation process.

## Stability analysis of candidate reference genes during asexual proliferation

Gene expression stability was ranked by geNorm, Comparative △*C_t_*, NormFinder, and BestKeeper, and the final ranking was calculated based on the aggregate mean of the ranking weights (Table 2). The gene stability rankings calculated by the four analysis methods differed, and the results of the four algorithms and the comprehensive ranking all showed that the four genes, *Rpb1*, *Gpd*, *Cox5*, and *Ubi*, had poor expression stability and were not suitable for use as reference genes. Results of three of the methods, geNorm, Comparative △*C_t_*, and NormFinder, indicated that better expression stability of *Tef1*, *Tub1*, *Tub2*, and *Tyr*, but BestKeeper results indicated that the stability ranking of *18s* was better than that of *Tub1*, *Tub2*, and *Tyr*. However, the expression of *18s* was significantly higher than that of other reference genes, and probably exceeded the confidence interval of the standard curve. Based on the rankings from each program, the comprehensive gene expression stability ranking (most stable to least stable) was calculated by RefFinder as: *Tef1*, *Tub1*, *Tub2*, *Tyr*, *18s*, *Actin*, *Rpb1*, *Gpd*, *Cox5*, and *Ubi*.

**Table 2. Stability analysis of candidate reference genes in asexual proliferation samples.**

| **Gene** | **geNorm** | **Comparative △*C_t_*** | | **NormFinder** | **BestKeeper ^4^** | | | **Comprehensive Stability value ^5^** | **Ranking ^6^** |
| --- | --- | --- | --- | --- | --- | --- | --- | --- | --- |
|  | **Stability value ^1^** | **Average of STDEV ^2^** | | **Stability value ^3^** | **SD** | | **CV** |  |  |
| *Tef1* | 0.317 | 2.51 | 0.15 | | | 0.39 | 2.25 | 1.86 | 1 |
| *Tub1* | 0.153 | 2.52 | 0.077 | | | 0.67 | 3.33 | 1.97 | 2 |
| *Tub2* | 0.256 | 2.52 | 0.141 | | | 0.56 | 2.5 | 2.63 | 3 |
| *Tyr* | 0.153 | 2.54 | 0.184 | | | 0.71 | 2.91 | 3.13 | 4 |
| *18s* | 0.457 | 2.55 | 0.304 | | | 0.48 | 5.48 | 4.16 | 5 |
| *Actin* | 0.638 | 2.69 | 0.255 | | | 0.54 | 2.37 | 4.82 | 6 |
| *Rpb1* | 0.913 | 3.15 | 1.427 | | | 0.99 | 4.02 | 7 | 7 |
| *Gpd* | 2.05 | 6.18 | 5.527 | | | 2.62 | 13.33 | 8 | 8 |
| *Cox5* | 2.962 | 6.54 | 5.952 | | | 2.85 | 13.33 | 9 | 9 |
| *Ubi* | 3.87 | 7.5 | 7.072 | | | 3.34 | 13.68 | 10 | 10 |

^1^ The stability value calculated by geNorm is the standard deviation of the expression variation of the two genes. A high stability value indicates low expression stability.

A high stability value indicates low expression stability, while a low stability value indicates high expression stability.

^2^ Comparative Δ*C_t_* method ranks candidate gene stability according to the repeatability of gene expression differences. A low Average of STDEV indicates high expression stability.

^3^ The stability value calculated by NormFinder. A high stability value indicates low expression stability.

^4^ BestKeeper analyzes expression stability by calculating cycle threshold (Ct) data variation. A low SD (standard deviation) and CV (Coefficient of Variance) value indicates high expression stability.

^5^ Comprehensive stability value assigns the appropriate weights to individual genes based on the ranking of each program and calculates the geometric mean of their weights for the overall final ranking.

^6^ Ranking assigned to each gene according to its comprehensive stability value.

## Stability analysis of candidate reference genes during fruiting body formed

During fruiting body development, the results of candidate reference gene expression stability calculated by geNorm, Comparative △*C_t_*, and NormFinder were similar (Table 3). *Tyr*, *Cox5*, *Gpd*, and *Actin* had better stability, whereas *Tef1*, *Tub1*, *Tub2*, and *Ubi* had poor stability, and the stability of *Ubi* calculated by geNorm exceeded the threshold of 1.5. In the BestKeeper calculation results, all genes exceeded the threshold except for *18s* and *Rpb1* (SD<1.0). However, based on the analysis results of other algorithms, we considered that the comprehensive stability ranking (most stable to least stable) was as follows: *Tyr*, *Cox5*, *Gpd*, *Actin*, *18s*, *Rpb1*, *Tef1*, *Tub1*, *Tub2*, and *Ubi*.

**Table 3. Stability analysis of candidate reference genes in fruiting body formed samples.**

| **Gene** | **geNorm** | **Comparative △*C_t_*** | | **NormFinder** | **BestKeeper ^4^** | | | **Comprehensive Stability value ^5^** | **Ranking ^6^** |
| --- | --- | --- | --- | --- | --- | --- | --- | --- | --- |
|  | **Stability value ^1^** | **Average of STDEV ^2^** | | **Stability value ^3^** | **SD** | | **CV** |  |  |
| *Tyr* | 0.689 | 1.42 | 0.307 | | | 1.09 | 4.22 | 1.86 | 1 |
| *Cox5* | 0.619 | 1.46 | 0.307 | | | 1.29 | 5 | 2.11 | 2 |
| *Gpd* | 0.619 | 1.5 | 0.394 | | | 1.38 | 5.62 | 3.13 | 3 |
| *Actin* | 0.658 | 1.5 | 0.31 | | | 1.28 | 4.9 | 3.22 | 4 |
| *18s* | 1.031 | 2.04 | 1.462 | | | 0.4 | 4.18 | 4.45 | 5 |
| *Rpb1* | 0.867 | 1.65 | 0.739 | | | 0.67 | 2.45 | 4.56 | 6 |
| *Tef1* | 0.757 | 1.53 | 0.717 | | | 1.43 | 7.28 | 5.44 | 7 |
| *Tub1* | 1.17 | 1.96 | 1.518 | | | 1.7 | 8.66 | 7.74 | 8 |
| *Tub2* | 1.302 | 2.17 | 1.789 | | | 2.34 | 10.48 | 9.24 | 9 |
| *Ubi* | 2.003 | 4.81 | 4.718 | | | 1.91 | 6.36 | 9.74 | 10 |

The footer is the same as Table 2

## Stability analysis of candidate reference genes under light treatment

Under light conditions, the three most stable expression genes calculated by geNorm, Comparative △*C_t_*, and NormFinder were *Tyr*, *Tef1*, and *Actin*, whereas the least stable genes were *Tub1*, *Tub2*, *Gpd*, and *Cox5*. Additionally, among the three genes with the most stable expression, there was little difference in stability value (Table 4). In BestKeeper, only *Rpb1*, *18s*, and *Tub1* met the screening conditions (SD<1.0). Based on the rankings from each program, the final comprehensive stability ranking (most stable to least stable) was *Tyr*, *Tef1*, *Actin*, *Rpb1*, *18s*, *Ubi*, *Tub1*, *Tub2*, *Gpd*, and *Cox5*.

**Table 4. Stability analysis of candidate reference genes in light treatment samples.**

| **Gene** | **geNorm** | **Comparative △*C_t_*** | | **NormFinder** | **BestKeeper ^4^** | | | **Comprehensive Stability value ^5^** | **Ranking ^6^** |
| --- | --- | --- | --- | --- | --- | --- | --- | --- | --- |
|  | **Stability value ^1^** | **Average of STDEV ^2^** | | **Stability value ^3^** | **SD** | | **CV** |  |  |
| *Tyr* | 0.579 | 1.59 | 0.289 | | | 1.01 | 3.99 | 1.68 | 1 |
| *Tef1* | 0.579 | 1.62 | 0.289 | | | 1.38 | 7.37 | 1.93 | 2 |
| *Actin* | 0.709 | 1.69 | 0.322 | | | 1.37 | 5.35 | 3.57 | 3 |
| *Rpb1* | 0.843 | 1.7 | 0.766 | | | 0.71 | 2.59 | 3.76 | 4 |
| *18s* | 0.924 | 1.91 | 1.172 | | | 0.52 | 5.43 | 3.83 | 5 |
| *Ubi* | 0.755 | 1.75 | 0.336 | | | 1.33 | 4.42 | 4.47 | 6 |
| *Tub1* | 1.061 | 2.05 | 1.466 | | | 0.98 | 5.19 | 5.66 | 7 |
| *Tub2* | 1.172 | 2.15 | 1.536 | | | 1.48 | 6.87 | 8 | 8 |
| *Gpd* | 1.793 | 4.1 | 3.813 | | | 2.41 | 10.43 | 9.24 | 9 |
| *Cox5* | 2.279 | 4.22 | 3.958 | | | 2.25 | 9.17 | 9.74 | 10 |

The footer is the same as Table 2

# Discussion

RT-qPCR is an important method for studying gene expression that can help eliminate experimental errors, and selecting a stably expressed reference gene to correct the results is necessary [28]. In this study, the asexual proliferation, fruiting body development, and effects of light on *O. sinensis* were recorded in detail. Additionally, we assessed the stability of 10 candidate reference genes under three different experimental conditions. These results provide a reference for studying the gene expression of *O. sinensis* under different models. Through this study, we found that the reference genes stably expressed by *O. sinensis* are not the same under different proliferation forms and treatment conditions, which further demonstrated the importance of selecting appropriate reference genes.

The formation process of Chinese cordyceps can be divided into two stages. One is the proliferation stage of *O. sinensis* in the hemolymph of *T. armoricanus* larvae. It is generally believed that, in this stage, *O. sinensis* mainly propagates asexually in the form of hyphal bodies [29]. At the subsequent stage, the hyphal bodies are induced by certain signal substances to transform by hyphae proliferation [30]. Our results revealed that, as the content of *O. sinensis* mycelium in the host hemolymph increased, the mycelium began clustering and caused the host larvae died and mummified. The second stage is the formation of fruiting bodies [31]. It is generally believed that, similar to mushrooms [32], the formation of fruiting bodies of *O. sinensis* is also the result of sexual mating, and asci will be formed in the middle parts of the fruiting bodies at the later stage of development [18]. Although the molecular mechanism of Chinese cordyceps formation has not been completely analyzed, as a key node, the dimorphic transition (from blastospores or hyphal bodies to mycelial pellets) [33] and the developmental regulation of fruiting bodies have attracted the attention of many scholars.

In this study, the asexual proliferation process of *O. sinensis* was simulated by liquid fermentation, and the formation of blastospores (similar to hyphal bodies), mycelia, and mycelial pellets was more accurately reproduced. Using different types of samples, we screened the stably expressed reference genes *Tef1* and *Tub1* during asexual proliferation. Although it could not be proven that the proliferation process of *O. sinensis* in liquid fermentation and host hemolymph was the same, the similar morphological changes can also provide a reference for studying the dimorphic transition of *O. sinensis*. During the fruiting body developmental stage, we collected samples from the mummified larva until the fruiting body matured and formed asci. By using fruiting body samples from various developmental stages, we determined that *Tyr* and *Cox5* represented stably expressed reference genes during the fruiting body development process.

During the process of asexual proliferation and fruiting body development, we found that light can affect *O. sinensis* color and ascus formation. Fungi can respond to light in various ways; for example, fungi can respond to different wavelengths of light through photoreceptor proteins, and can also regulate the expression of downstream genes through light-induced intracellular ROS and NOS [34,35]. In *Cordyceps militaris*, researchers usually use *Rbp1* as an internal reference gene when studying the regulation of light in fungal gene expression [36]. However, in this study, we found that *Rbp1* is not a stable reference gene for *O. sinensis* response to light. In a previous study, the researchers used *18s* as a reference gene to explore the expression pattern of the blue light receptor *OsWC1* in *O. Sinensis* [19]. It is generally believed that *18s* expression is stable and not easily affected by other conditions [37], but the expression level of *18s* is very high compared with other genes, and it is likely to exceed the confidence interval of the standard curve [38]. In this study, we determined that the reference genes *Tyr* and *Tef1* are more stable than *18s* and *Rbp1*, which can be used for follow-up studies of *O. sinensis* response to light.

# Conclusions

To accurately determine gene expression, some researchers suggest that two or more stable reference genes should be used to normalize expression levels. In this study, we used four methods (geNorm, Comparative △*C_t_*, NormFinder, and BestKeeper) to evaluate the stability value of reference genes, and used RefFinder to integrate the stability ranking of each method to comprehensively evaluate the stable reference genes of *O. sinensis* in asexual proliferation, fruiting body development, and light-induced conditions. We found that the stable reference genes of *O. sinensis* differed under different conditions, and this study provides a reference for selecting suitable stable reference genes under different conditions.

# References

1.	Li SP, Li P, Dong TT, Tsim KW. Anti-oxidation activity of different types of natural *Cordyceps* *sinensis* and cultured Cordyceps mycelia. Phytomedicine. 2001;8: 207–212. doi:10.1078/0944-7113-00030

2.	Yue K, Ye M, Zhou Z, Sun W, Lin X. The genus *Cordyceps*: a chemical and pharmacological review. J Pharm Pharmacol. 2013;65: 474–493. doi:10.1111/j.2042-7158.2012.01601.x

3.	Li L, Song A, Yin J, Siu K, Wong W, Wu J. Anti-inflammation activity of exopolysaccharides produced by a medicinal fungus *Cordyceps* *sinensis* Cs-HK1 in cell and animal models. Int J Biol Macromol. 2020;149: 1042–1050. doi:10.1016/j.ijbiomac.2020.02.022

4.	Lu Q, Li C, Chen W, Shi Z, Zhan R, He R. Clinical Efficacy of Jinshuibao Capsules Combined with Angiotensin Receptor Blockers in Patients with Early Diabetic Nephropathy: A Meta-Analysis of Randomized Controlled Trials. Evid Based Complement Alternat Med. 2018;2018: 6806943. doi:10.1155/2018/6806943

5.	Liu Y, Li Q-Z, Li L-D-J, Zhou X-W. Immunostimulatory effects of the intracellular polysaccharides isolated from liquid culture of *Ophiocordyceps* *sinensis* (Ascomycetes) on RAW264.7 cells via the MAPK and PI3K/Akt signaling pathways. J Ethnopharmacol. 2021;275: 114130. doi:10.1016/j.jep.2021.114130

6.	Shrestha UB, Bawa KS. Impact of climate change on potential distribution of Chinese caterpillar fungus (*Ophiocordyceps* *sinensis*) in Nepal Himalaya. PLoS One. 2014;9: e106405. doi:10.1371/journal.pone.0106405

7.	Tao Z, Cao L, Zhang Y, Ye Y, Han R. Laboratory Rearing of *Thitarodes* *armoricanus* and Thitarodes *jianchuanensis* (Lepidoptera: Hepialidae), Hosts of the Chinese Medicinal Fungus *Ophiocordyceps* *sinensis* (Hypocreales: Ophiocordycipitaceae). J Econ Entomol. 2016;109: 176–181. doi:10.1093/jee/tov319

8.	Yue K, Ye M, Lin X, Zhou Z. The artificial cultivation of medicinal Caterpillar Fungus, *Ophiocordyceps* *sinensis* (Ascomycetes): a review. Int J Med Mushrooms. 2013;15: 425–434. doi:10.1615/intjmedmushr.v15.i5.10

9.	Tao H, Cao L, Han R. Influence of medium components and plant growth regulators on artificial production of fruiting bodies of *Ophiocordyceps* *sinensis*. Journal of Environmental Entomology. 2020;42: 274–281. doi:10.3969/j.issn.1674－0858.2020.02.4

10.	Qin Q, Zhou G, Zhang H, Meng Q, Zhang J, Wang H, et al. Obstacles and approaches in artificial cultivation of Chinese cordyceps. Mycology. 2018;9: 7–9. doi:10.1080/21501203.2018.1442132

11.	Zhang S, Cen K, Liu Y, Zhou X, Wang C. Metatranscriptomics analysis of the fruiting caterpillar fungus collected from the Qinghai-Tibetan plateau. SCIENTIA SINICA Vitae. 2018;48: 562–570. doi:10.1360/N052017-00253

12.	Deng X, Wei Q, He Y, Shi P, Xing K, Chen S. Color formation in artificially cultivated *Ophiocordyceps* *sinensis*. Chinese Journal of Applied Entomology. 2021;58: 1339–1346. doi:10.7679/j.issn.2095-1353.2021.134

13.	Li L, Luo H, Hu M. Research on the Artificial Cultivation of the Conidia by *Ophiocordyceps* *Sinensis*. Food Research And Development. 2016;37: 187–190. doi:10.3969/j.issn.1005-6521.2016.03.047

14.	Zhong X, Gu L, Wang H, Lian D, Zheng Y, Zhou S, et al. Profile of *Ophiocordyceps* *sinensis* transcriptome and differentially expressed genes in three different mycelia, sclerotium and fruiting body developmental stages. Fungal Biol. 2018;122: 943–951. doi:10.1016/j.funbio.2018.05.011

15.	Tong X, Zhang H, Wang F, Xue Z, Cao J, Peng C, et al. Comparative transcriptome analysis revealed genes involved in the fruiting body development of *Ophiocordyceps* *sinensis*. PeerJ. 2020;8: e8379. doi:10.7717/peerj.8379

16.	Tong X, Wang F, Zhang H, Bai J, Dong Q, Yue P, et al. iTRAQ-based comparative proteome analyses of different growth stages revealing the regulatory role of reactive oxygen species in the fruiting body development of *Ophiocordyceps* *sinensis*. PeerJ. 2021;9: e10940. doi:10.7717/peerj.10940

17.	Liu Y, Xiao K, Wang Z, Wang S, Xu F. Comparison of metabolism substances in *Cordyceps* *sinensis* and *Cordyceps* *militaris* cultivated with tussah pupa based on LC-MS. J Food Biochem. 2021;45: e13735. doi:10.1111/jfbc.13735

18.	Xiang L, Li Y, Zhu Y, Luo H, Li C, Xu X, et al. Transcriptome analysis of the *Ophiocordyceps* *sinensis* fruiting body reveals putative genes involved in fruiting body development and cordycepin biosynthesis. Genomics. 2014;103: 154–159. doi:10.1016/j.ygeno.2014.01.002

19.	Yang T, Xiong W, Dong C. Cloning and analysis of the Oswc-1 gene encoding a putative blue light photoreceptor from *Ophiocordyceps* *sinensis*. Mycoscience. 2014;55: 241–245. doi:10.1016/j.myc.2013.09.003

20.	Dong C, Yao Y. Isolation, characterization of melanin derived from *Ophiocordyceps* *sinensis*, an entomogenous fungus endemic to the Tibetan Plateau. J Biosci Bioeng. 2012;113: 474–479. doi:10.1016/j.jbiosc.2011.12.001

21.	Vandesompele J, De Preter K, Pattyn F, Poppe B, Van Roy N, De Paepe A, et al. Accurate normalization of real-time quantitative RT-PCR data by geometric averaging of multiple internal control genes. Genome Biol. 2002;3: RESEARCH0034. doi:10.1186/gb-2002-3-7-research0034

22.	Huggett J, Dheda K, Bustin S, Zumla A. Real-time RT-PCR normalisation; strategies and considerations. Genes Immun. 2005;6: 279–284. doi:10.1038/sj.gene.6364190

23.	Li X, Wang F, Liu Q, Li Q, Qian Z, Zhang X, et al. Developmental transcriptomics of Chinese cordyceps reveals gene regulatory network and expression profiles of sexual development-related genes. BMC Genomics. 2019;20: 337. doi:10.1186/s12864-019-5708-z

24.	Andersen CL, Jensen JL, Ørntoft TF. Normalization of Real-Time Quantitative Reverse Transcription-PCR Data: A Model-Based Variance Estimation Approach to Identify Genes Suited for Normalization, Applied to Bladder and Colon Cancer Data Sets. Cancer Research. 2004;64: 5245–5250. doi:10.1158/0008-5472.CAN-04-0496

25.	Pfaffl MW, Tichopad A, Prgomet C, Neuvians TP. Determination of stable housekeeping genes, differentially regulated target genes and sample integrity: BestKeeper--Excel-based tool using pair-wise correlations. Biotechnol Lett. 2004;26: 509–515. doi:10.1023/b:bile.0000019559.84305.47

26.	Silver N, Best S, Jiang J, Thein SL. Selection of housekeeping genes for gene expression studies in human reticulocytes using real-time PCR. BMC Mol Biol. 2006;7: 33. doi:10.1186/1471-2199-7-33

27.	Xie F, Xiao P, Chen D, Xu L, Zhang B. miRDeepFinder: a miRNA analysis tool for deep sequencing of plant small RNAs. Plant Mol Biol. 2012. doi:10.1007/s11103-012-9885-2

28.	Llanos A, François JM, Parrou J-L. Tracking the best reference genes for RT-qPCR data normalization in filamentous fungi. BMC Genomics. 2015;16: 71. doi:10.1186/s12864-015-1224-y

29.	Li M, Meng Q, Zhang H, Ni R, Zhou G, Zhao Y, et al. Vegetative development and host immune interaction of *Ophiocordyceps* *sinensis* within the hemocoel of the ghost moth larva, *Thitarodes* *xiaojinensis*. J Invertebr Pathol. 2020;170: 107331. doi:10.1016/j.jip.2020.107331

30.	Ren S-Y, Yao Y-J. Evaluation of nutritional and physical stress conditions during vegetative growth on conidial production and germination in *Ophiocordyceps* *sinensis*. FEMS Microbiol Lett. 2013;346: 29–35. doi:10.1111/1574-6968.12190

31.	Cao L, Ye Y, Han R. Fruiting Body Production of the Medicinal Chinese Caterpillar Mushroom, *Ophiocordyceps* *sinensis* (Ascomycetes), in Artificial Medium. Int J Med Mushrooms. 2015;17: 1107–1112. doi:10.1615/intjmedmushrooms.v17.i11.110

32.	Pöggeler S, Nowrousian M, Teichert I, Beier A, Kück U. Fruiting-Body Development in Ascomycetes. In: Anke T, Schüffler A, editors. Physiology and Genetics: Selected Basic and Applied Aspects. Cham: Springer International Publishing; 2018. pp. 1–56. doi:10.1007/978-3-319-71740-1_1

33.	Liu G, Cao L, Qiu X, Han R. Quorum Sensing Activity and Hyphal Growth by External Stimuli in the Entomopathogenic Fungus *Ophiocordyceps* *sinensis*. Insects. 2020;11: 205. doi:10.3390/insects11040205

34.	Pengkit A, Jeon SS, Son SJ, Shin JH, Baik KY, Choi EH, et al. Identification and functional analysis of endogenous nitric oxide in a filamentous fungus. Sci Rep. 2016;6: 30037. doi:10.1038/srep30037

35.	Zhao Y, Lim J, Xu J, Yu J-H, Zheng W. Nitric oxide as a developmental and metabolic signal in filamentous fungi. Mol Microbiol. 2020;113: 872–882. doi:10.1111/mmi.14465

36.	Lian T, Yang T, Liu G, Sun J, Dong C. Reliable reference gene selection for *Cordyceps* *militaris* gene expression studies under different developmental stages and media. FEMS Microbiol Lett. 2014;356: 97–104. doi:10.1111/1574-6968.12492

37.	Jin K, Zhang Y, Fang W, Luo Z, Zhou Y, Pei Y. Carboxylate transporter gene JEN1 from the entomopathogenic fungus *Beauveria* *bassiana* is involved in conidiation and virulence. Appl Environ Microbiol. 2010;76: 254–263. doi:10.1128/AEM.00882-09

38.	Zhou Y-H, Zhang Y-J, Luo Z-B, Fan Y-H, Tang G-R, Liu L-J, et al. Selection of optimal reference genes for expression analysis in the entomopathogenic fungus *Beauveria* *bassiana* during development, under changing nutrient conditions, and after exposure to abiotic stresses. Appl Microbiol Biotechnol. 2012;93: 679–685. doi:10.1007/s00253-011-3561-3

# Supporting information

**S1 Fig. Melting curves and standard curve of candidate reference genes.** Melting curves were analyzed by LightCycler® 96 Software 1.1 (Roche). The standard curve was amplified from 10-fold diluted standard plasmid.
