# Supplementary material for: Stable reference gene selection for Ophiocordyceps sinensis gene expression studies under different developmental stages and light-induced conditions
Source: PLoS One. 2023 Apr 20;18(4):e0284486. doi: 10.1371/journal.pone.0284486 (PMC10118168; doi:10.1371/journal.pone.0284486)
Supplement: Attachment — Submitted filename: Review comments for the Manuscript.docx [file pone.0284486.s003.docx]

**Review comments for the Manuscript entitled**

**“**Stable reference gene selection for *Ophiocordyceps sinensis* gene expression studies under different developmental stages and light-induced conditions”

1. Abstract

The background is too short. It should be explain on the main objective of the study. In the methods and results of the abstract, please mention the results that has mentioned in the methods. More information about the highly significant observations should be clarified in the results. You should provide conclusion with focusing on the innovations related to be in a harmony with the title of the manuscript and the objectives of your study.

2. Introduction

- Generally, introduction section needs several enhancements and error checks. I think the language should be rechecked by an English expert for grammar and typos.

- You should focus on the objectives of your study. Introduce why your study are important with recent references.

- The best reference is within a period of 5 years (2018-2022)

- The introduction section in this manuscript has been written non-comprehensively. Author should outline the current situation and also evaluate the current situation (advantages/ disadvantages) and identify the gap.

- Problem statement is not very strong and clear. It needs a comprehensive explanation.

- In the last paragraph, please add a couple of sentences to highlight the “innovative” aspect of the paper (added value in this field?) …what has been examined for very first time/or in more depth in this research area by your work presented in this manuscript?

- The length of each paragraph needs to be harmonized.

Please ensure that the scientific name is written correctly throughout this manuscript

3. Method

- Please explain each method that has been used in this study in detail.

- Please include the references for your method.

 4. Results

- This part of the result is displayed well; it needs to be rewritten and organized systematically so that the reader can better understand what the author wants to explain.

Misspelling the word is a minor mistake. Please take a look at the whole manuscript and correct it accordingly.

- Please include the Figure.

- Do not change the resolution of the figure because it will affect the quality of the figure. Do not change the resolution of the figure because it will affect the quality of the figure and caused the culture plate to be not the same size.

5. Discussion

- Generally, this section needs to be supplemented with more other literature review papers. I think you should discuss the innovations and more clarifying why your study in this section in more details.

- Every statement needs to be supported with the correct reference source. It is strictly necessary to use the latest reference sources. The authors needs to update the reference sources used. Make sure it is up to date.

- Each result needs to be discussed comprehensively and strengthened by referring to the previous studies

 6. Conclusion: More details about your observations by showing some of this valuable study significations point by point should be more clarified.

7. References

A number or references need some formatting changes. Please ensure that you follow PLOS one guidelines for presenting name of authors/abbreviation of journals/year of publication in bold/volume journals in italics etc
