# Supplementary material for: Stable reference gene selection for Ophiocordyceps sinensis gene expression studies under different developmental stages and light-induced conditions
Source: PLoS One. 2023 Apr 20;18(4):e0284486. doi: 10.1371/journal.pone.0284486 (PMC10118168; doi:10.1371/journal.pone.0284486)
Supplement: Attachment — Submitted filename: Response to Editor and Reviewers-0302.docx [file pone.0284486.s004.docx]

Dear Editor and Reviewers,

Thanks for your letter and comments concerning our manuscript (PONE-D-22-31224). The comments are all valuable and very helpful for revising and improving our paper, as well as the important guiding significance to our research.

We have uploaded the revised manuscript. The revised portions are marked in blue in the version of the manuscript with the changes highlighted. The responses to the reviewer’s comments are as follows.

Thank you very much for all your help and looking forward to hearing from you.

Sincerely,

Chunfeng Li，PhD

State Key Laboratory of Silkworm Genome Biology, Southwest University, Chongqing, 400715, China.

licf @swu.edu.cn

**Response to Editor and Reviewers**

Thank you much for your kind and constructive comments which served as a welcome guideline to improve the manuscript. According to your suggestion, we have revised our manuscript carefully. We read the manuscript carefully and provide a point-by-point response to the reviewer’s comments.

**Response to Editor**

*1. Abstract*

*The background is too short. It should be explain on the main objective of the study. In the methods and results of the abstract, please mention the results that has mentioned in the methods. More information about the highly significant observations should be clarified in the results. You should provide conclusion with focusing on the innovations related to be in a harmony with the title of the manuscript and the objectives of your study.*

Response: Thank you for your comment. We added the background and purpose of the study in line 19-25 of the Abstract, as follows,

*The formation process of Chinese cordyceps includes two parts: asexual proliferation (Ophiocordyceps sinensis proliferates in the hemolymph of Thitarodes armoricanus larvae) and sexual development (formation and development of fruiting bodies). Therefore, validation of reference genes under different development stages and experimental conditions is crucial for RT-qPCR analysis. However, there is no report on stable reference genes at the development stage of O. sinensis fruiting body.*

*2. Introduction*

*- Generally, introduction section needs several enhancements and error checks. I think the language should be rechecked by an English expert for grammar and typos.*

*- You should focus on the objectives of your study. Introduce why your study are important with recent references.*

*- The best reference is within a period of 5 years (2018-2022)*

*- The introduction section in this manuscript has been written non-comprehensively. Author should outline the current situation and also evaluate the current situation (advantages/ disadvantages) and identify the gap.*

*- Problem statement is not very strong and clear. It needs a comprehensive explanation.*

*- In the last paragraph, please add a couple of sentences to highlight the “innovative” aspect of the paper (added value in this field?) …what has been examined for very first time/or in more depth in this research area by your work presented in this manuscript?*

*- The length of each paragraph needs to be harmonized.*

*Please ensure that the scientific name is written correctly throughout this manuscript*

Response: Thank you for your comment. In the Introduction, we have made the following changes respectively,

1. We thank the reviewer for the comments. The manuscript has been re-edited by the language-editing professionals.
2. After introducing the background and research status of Chinese cordyceps, we added that we mainly focus on the formation and development of *O. sinensis* fruiting body, please see line 61-67.
3. We summarized the importance of stable reference genes in RT-qPCR, and the necessity of retesting the stability of reference genes in different experimental models. However, there is no report on the research of reference genes during the development of Chinese cordyceps fruiting body, which shows the importance of our research. Please see line 72-83.
4. In the last paragraph, we mainly introduce the innovation of this study. We introduced the samples with different phenotypes used in this study. In addition to the development stage, our samples also included the effect of light stress on the proliferation of *O. sinensis* and the development of fruiting bodies. This is the first study using samples with various time periods and light stress. Comprehensive sampling can provide appropriate internal reference genes for subsequent researchers to study gene expression. Please see line 84-96.

At the same time, we updated some references to the latest five years, as follows,

| **Old Reference No.** | **Author and year** | **New Reference No.** | **Author and year** |
| --- | --- | --- | --- |
| 1,2 | (Li *et al.* 2001; Yue *et al.* 2013b) | 1 | (Tong and Guo 2022) |
| 7,8 | (Yue *et al.* 2013a; Tao *et al.* 2016) | 5,6 | (Zhang *et al.* 2018; Liu *et al.* 2019) |
| 13 | (Li *et al.* 2016) | 11 | (Xie *et al.* 2022) |
| 18 | (Xiang *et al.* 2014) | 16 | (Zhao *et al.* 2020) |
| 19 | (Yang *et al.* 2014) | 11 | (Xie *et al.* 2022) |
| 21 | (Vandesompele *et al.* 2002) | 18,19 | (Zhao *et al.* 2018; Fu *et al.* 2022) |
| 22 | (Huggett *et al.* 2005) | 20 | (Zhu *et al.* 2021) |

Fu, N., Li, J., Wang, M., Ren, L., Zong, S., and Luo, Y. 2022. Identification and Validation of Reference Genes for Gene Expression Analysis in Different Development Stages of *Amylostereum* *areolatum*. Front Microbiol 12: 827241. doi:10.3389/fmicb.2021.827241.

Huggett, J., Dheda, K., Bustin, S., and Zumla, A. 2005. Real-time RT-PCR normalisation; strategies and considerations. Genes Immun 6: 279–284. Nature Publishing Group. doi:10.1038/sj.gene.6364190.

Li, L., Luo, H., and Hu, M. 2016. Research on the Artificial Cultivation of the Conidia by *Ophiocordyceps* *Sinensis*. Food Research And Development 37: 187–190. doi:10.3969/j.issn.1005-6521.2016.03.047.

Li, S.P., Li, P., Dong, T.T., and Tsim, K.W. 2001. Anti-oxidation activity of different types of natural *Cordyceps* *sinensis* and cultured *Cordyceps* *mycelia*. Phytomedicine 8: 207–212. doi:10.1078/0944-7113-00030.

Liu, G., Han, R., and Cao, L. 2019. Artificial Cultivation of the Chinese Cordyceps From Injected Ghost Moth Larvae. Environ Entomol 48: 1088–1094. doi:10.1093/ee/nvz099.

Tao, Z., Cao, L., Zhang, Y., Ye, Y., and Han, R. 2016. Laboratory Rearing of *Thitarodes* *armoricanus* and *Thitarodes* *jianchuanensis* (Lepidoptera: Hepialidae), Hosts of the Chinese Medicinal Fungus *Ophiocordyceps* *sinensis* (Hypocreales: Ophiocordycipitaceae). J Econ Entomol 109: 176–181. doi:10.1093/jee/tov319.

Tong, X., and Guo, J. 2022. High Throughput Identification of the Potential Antioxidant Peptides in *Ophiocordyceps* *sinensis*. Molecules 27: 438. doi:10.3390/molecules27020438.

Vandesompele, J., De Preter, K., Pattyn, F., Poppe, B., Van Roy, N., De Paepe, A., and Speleman, F. 2002. Accurate normalization of real-time quantitative RT-PCR data by geometric averaging of multiple internal control genes. Genome Biol 3: RESEARCH0034. doi:10.1186/gb-2002-3-7-research0034.

Xiang, L., Li, Y., Zhu, Y., Luo, H., Li, C., Xu, X., Sun, C., Song, J., Shi, L., He, L., Sun, W., and Chen, S. 2014. Transcriptome analysis of the *Ophiocordyceps* *sinensis* fruiting body reveals putative genes involved in fruiting body development and cordycepin biosynthesis. Genomics 103: 154–159. doi:10.1016/j.ygeno.2014.01.002.

Xie, F., He, L., Su, Q.J., Xia, Y., Zhou, G., and Chen, Z.H. 2022. Transcriptome Sequencing to Identify Candidate Genes for Mycelium Period Metabolism of Chinese Caterpillar Mushroom, *Ophiocordyceps* *sinensis* (Ascomycetes), Exposed to Light Treatment. Int J Med Mushrooms 24: 81–97. doi:10.1615/IntJMedMushrooms.2022044445.

Yang, T., Xiong, W., and Dong, C. 2014. Cloning and analysis of the *Oswc*-1 gene encoding a putative blue light photoreceptor from *Ophiocordyceps* *sinensis*. Mycoscience 55: 241–245. doi:10.1016/j.myc.2013.09.003.

Yue, K., Ye, M., Lin, X., and Zhou, Z. 2013a. The artificial cultivation of medicinal Caterpillar Fungus, *Ophiocordyceps* *sinensis* (Ascomycetes): a review. Int J Med Mushrooms 15: 425–434. doi:10.1615/intjmedmushr.v15.i5.10.

Yue, K., Ye, M., Zhou, Z., Sun, W., and Lin, X. 2013b. The genus *Cordyceps*: a chemical and pharmacological review. J Pharm Pharmacol 65: 474–493. doi:10.1111/j.2042-7158.2012.01601.x.

Zhang, X., Liu, Q., Zhou, W., Li, P., Alolga, R.N., Qi, L.-W., and Yin, X. 2018. A comparative proteomic characterization and nutritional assessment of naturally- and artificially-cultivated *Cordyceps* *sinensis*. J Proteomics 181: 24–35. doi:10.1016/j.jprot.2018.03.029.

Zhao, X., Yang, H., Chen, M., Song, X., Yu, C., Zhao, Y., and Wu, Y. 2018. Reference Gene Selection for Quantitative Real-Time PCR of Mycelia from *Lentinula* *edodes* under High-Temperature Stress. Biomed Res Int 2018: 1670328. doi:10.1155/2018/1670328.

Zhao, Y., Zhang, J., Meng, Q., Zhang, H., Zhou, G., Li, M., Wu, P., Shu, R., Gao, X., Guo, L., Tong, Y., Cheng, L., Guo, L., Chen, C., and Qin, Q. 2020. Transcriptomic analysis of the orchestrated molecular mechanisms underlying fruiting body initiation in Chinese cordyceps. Gene 763: 145061. doi:10.1016/j.gene.2020.145061.

Zhu, H., Ma, Y., and Guo, Q. 2021. Expression stability of internal reference gene in response to *Trichoderma* *polysporum* infection in *Avena* *fatua* L. Curr Genet 67: 909–918. doi:10.1007/s00294-021-01200-4.

We are sorry about that the following five references have not been replaced. The research of Dong and Yao is the first and only report on *O.* *sinensis* melanin directly. Although there are other studies on the color of Chinese cordyceps, they do not directly focus on melanin. In this study, we found that Chinese cordyceps also has a black phenotype under light stress, which attracted our interest. In another article, we studied the relationship between light and melanin in detail, and found that *O.* *sinensis* respond to light signals through photoreceptor proteins (WC1 and WC2), and then regulate the transcription of downstream target genes (unpublished). The research of Vandesompele *et al.*, Pfaffl *et al.*, Andersen *et al.* and Silver *et al.* are reports on the methods for evaluating the stability of reference genes, which has been cited many times as a classical method. In this study, we also used their methods. So, although these five references were published at least 10 years ago, we believe that they are directly related to our research and we still cite these articles.

Andersen, C.L., Jensen, J.L., and Ørntoft, T.F. 2004. Normalization of Real-Time Quantitative Reverse Transcription-PCR Data: A Model-Based Variance Estimation Approach to Identify Genes Suited for Normalization, Applied to Bladder and Colon Cancer Data Sets. Cancer Research 64: 5245–5250. doi:10.1158/0008-5472.CAN-04-0496.

Dong, C., and Yao, Y. 2012. Isolation, characterization of melanin derived from *Ophiocordyceps* *sinensis*, an entomogenous fungus endemic to the Tibetan Plateau. J Biosci Bioeng 113: 474–479. doi:10.1016/j.jbiosc.2011.12.001.

Pfaffl, M.W., Tichopad, A., Prgomet, C., and Neuvians, T.P. 2004. Determination of stable housekeeping genes, differentially regulated target genes and sample integrity: BestKeeper--Excel-based tool using pair-wise correlations. Biotechnol Lett 26: 509–515. doi:10.1023/b:bile.0000019559.84305.47.

Silver, N., Best, S., Jiang, J., and Thein, S.L. 2006. Selection of housekeeping genes for gene expression studies in human reticulocytes using real-time PCR. BMC Mol Biol 7: 33. doi:10.1186/1471-2199-7-33.

Vandesompele, J., De Preter, K., Pattyn, F., Poppe, B., Van Roy, N., De Paepe, A., and Speleman, F. 2002. Accurate normalization of real-time quantitative RT-PCR data by geometric averaging of multiple internal control genes. Genome Biol 3: RESEARCH0034. doi:10.1186/gb-2002-3-7-research0034.

*3. Method*

*- Please explain each method that has been used in this study in detail.*

*- Please include the references for your method.*

Response: Thank you for your comment. In the Method section, we have added the principles of various methods used to calculate the stability of reference genes, and their references. Please see line 149-160.

*4. Results*

*- This part of the result is displayed well; it needs to be rewritten and organized systematically so that the reader can better understand what the author wants to explain.*

*Misspelling the word is a minor mistake. Please take a look at the whole manuscript and correct it accordingly.*

*- Please include the Figure.*

*- Do not change the resolution of the figure because it will affect the quality of the figure. Do not change the resolution of the figure because it will affect the quality of the figure and caused the culture plate to be not the same size.*

Response: Thank you for your comment. For the comments on “*it needs to be rewritten and organized systematically so that the reader can better understand what the author wants to explain”*, we describe the results of each method separately, and add the evaluation of minimal number of genes for qRT-PCR normalization in geNorm section (Figure 3 in MS).

We rechecked and corrected the spelling and grammar errors. We uploaded the original high-resolution figures through the submission system.

*5. Discussion*

*- Generally, this section needs to be supplemented with more other literature review papers. I think you should discuss the innovations and more clarifying why your study in this section in more details.*

*- Every statement needs to be supported with the correct reference source. It is strictly necessary to use the latest reference sources. The authors needs to update the reference sources used. Make sure it is up to date.*

*- Each result needs to be discussed comprehensively and strengthened by referring to the previous studies*

Response: Thank you for your comments. In the discussion section, we have added the contribution and innovation of this study to the research of Chinese cordyceps, and also added references to relevant literature. References also cite the latest relevant articles as far as possible. Please see line 335-347.

*6. Conclusion: More details about your observations by showing some of this valuable study significations point by point should be more clarified.*

 Response: Thank you for your comment. We rewrote the conclusion section, summarized the main work and achievements of this study, and emphasized the value and significance of this study. Please see line 366-376.

*7. References*

*A number or references need some formatting changes. Please ensure that you follow PLOS one guidelines for presenting name of authors/abbreviation of journals/year of publication in bold/volume journals in italics etc*

Response: Thank you for your comment. We revised the format of the References according to the requirements of PLOS one guidelines (<https://journals.plos.org/plosone/s/submission-guidelines#loc-references>).

**Journal Requirements:**

*When submitting your revision, we need you to address these additional requirements.*

*1. Please ensure that your manuscript meets PLOS ONE's style requirements, including those for file naming. The PLOS ONE style templates can be found at*

*https://journals.plos.org/plosone/s/file?id=wjVg/PLOSOne_formatting_sample_main_body.pdf and*

*https://journals.plos.org/plosone/s/file?id=ba62/PLOSOne_formatting_sample_title_authors_affiliations.pdf*

Response: Thank you for your comment. We revised the format of the MS and file name according to the requirements of PLOS one guidelines.

*2. We note that the grant information you provided in the ‘Funding Information’ and ‘Financial Disclosure’ sections do not match.*

*When you resubmit, please ensure that you provide the correct grant numbers for the awards you received for your study in the ‘Funding Information’ section.*

Response: We are very sorry to make this mistake. Our study was funded by the following funding. We also uploaded the information of the fund to the submission system.

Fundamental Research Funds for the Central Universities to Southwest University

Award Number: XDJK2018AA001

Recipient: Guoqing Pan

*3. Thank you for stating the following financial disclosure:*

*“The authors received no specific funding for this work. No Funding”*

*At this time, please address the following queries:*

*a) Please clarify the sources of funding (financial or material support) for your study. List the grants or organizations that supported your study, including funding received from your institution.*

*b) State what role the funders took in the study. If the funders had no role in your study, please state: “The funders had no role in study design, data collection and analysis, decision to publish, or preparation of the manuscript.”*

*c) If any authors received a salary from any of your funders, please state which authors and which funders.*

*d) If you did not receive any funding for this study, please state: “The authors received no specific funding for this work.”*

*Please include your amended statements within your cover letter; we will change the online submission form on your behalf.*

Response: We are very sorry to make this mistake. We chose the wrong choice when filling in the submission system, our study was funded by the funding: Fundamental Research Funds for the Central Universities to Southwest University

The funders had no role in study design, data collection and analysis, decision to publish, or preparation of the manuscript.

The authors declare no conflict of interest.

*4. In your Data Availability statement, you have not specified where the minimal data set underlying the results described in your manuscript can be found. PLOS defines a study's minimal data set as the underlying data used to reach the conclusions drawn in the manuscript and any additional data required to replicate the reported study findings in their entirety. All PLOS journals require that the minimal data set be made fully available. For more information about our data policy, please see http://journals.plos.org/plosone/s/data-availability.*

*Upon re-submitting your revised manuscript, please upload your study’s minimal underlying data set as either Supporting Information files or to a stable, public repository and include the relevant URLs, DOIs, or accession numbers within your revised cover letter. For a list of acceptable repositories, please see http://journals.plos.org/plosone/s/data-availability#loc-recommended-repositories. Any potentially identifying patient information must be fully anonymized.*

*Important: If there are ethical or legal restrictions to sharing your data publicly, please explain these restrictions in detail. Please see our guidelines for more information on what we consider unacceptable restrictions to publicly sharing data: http://journals.plos.org/plosone/s/data-availability#loc-unacceptable-data-access-restrictions. Note that it is not acceptable for the authors to be the sole named individuals responsible for ensuring data access.*

*We will update your Data Availability statement to reflect the information you provide in your cover letter.*

Response: Thank you for your comment. The all data used in this study are shown in MS and Supporting information.

**Additional Editor Comments:**

*The manuscript "Stable reference gene selection for Ophiocordyceps sinensis gene expression studies under different developmental stages and light-induced conditions" submitted by Chaoqun Tong et al is good work but it lack novelties. Similar kind of articles using Cordyceps sinensis and other species have been published in different journals. I can quote some of the articles here*

*1. SU Qiang-Jun, XIA Ying-Xia, XIE Fang, Uwitugabiye VESTINE, CHEN Zhao-He, ZHOU Gang. Screening of the reference genes for qRT-PCR analysis of gene expression in Ophiocordyceps sinensis[J].MYCOSYSTEMA, 2021, 40(7): 1712-1722.*

*2. Gangzheng Wang, Huijiao Cheng, Min Li, Chenghua Zhang, Wangqiu Deng, Taihui Li, Selection and validation of reliable reference genes for Tolypocladium guangdongense gene expression analysis under differentially developmental stages and temperature stresses,*

*Gene, Volume 734, 2020, 144380, ISSN 0378-1119, https://doi.org/10.1016/j.gene.2020.144380.*

*3. Tiantian Lian, Tao Yang, Guijun Liu, Junde Sun, Caihong Dong, Reliable reference gene selection for Cordyceps militaris gene expression studies under different developmental stages and media, FEMS Microbiology Letters, Volume 356, Issue 1, July 2014, Pages 97–104, https://doi.org/10.1111/1574-6968.12492*

*How will you justify the novelty of work when such work has been done by several authors on the same fungi.*

Response: Thank you for your comment. Many studies have proved that the expression of common housekeeping genes will change due to different species and experimental treatments, and there were no known stable reference gene applicable to all conditions. Therefore, many researchers have re-selected and verified stable reference genes in corresponding experimental models. In *Cerrena* *unicolor*, the expression levels of seven candidate reference genes were studied at different growth phases, under various induction conditions, and with a range of carbon/nitrogen ratios and carbon and nitrogen sources [1]. Similarly, as early as 2012, stable reference genes of *Beauveria bassiana* at different development stages and under various nutritional and stress conditions have been reported [2]. Subsequently, stable reference genes under different conditions were reported in *Volvariella* *volvacea* [3], *Amylostereum* *areolatum* [4] and *Lentinula* *edodes* [5]. Therefore, for a different species (*O. sinensis* in our study), the stability of reference genes needs to be re-evaluated.

Further, *O. sinensis* is a kind of low-temperature fungus. The optimum temperature for its growth is 16 ℃. The formation and development of fruiting body takes several months (as described in the results in MS), so it is very different from other edible fungi. It would be imprecise to directly use the reference genes of other fungal development processes in the study of *O. sinensis*. During the development of *C.* *militaris* fruiting body development, the most stable reference gene is *rpb1* [7], and our study found that *Try* and *Cox5* are considered to be the best reference genes in *O. sinensis* fruiting body development. Which confirms our conjecture that the development process of *O. sinensis* and *C.* *militaris* is different, and the selection of reference genes needs to be screened again.

So far, there is only one report (the article 1 you quoted above) on the research of reference genes in *O. sinensis* [6]. Their research mainly focused on the stability of reference genes in the asexually proliferated *O. sinensis* in solid medium (Figure1 below), and the results have good guiding value for the solid fermentation of *O. sinensis*. But we focus more on the formation process of Chinese cordyceps, including the multiplication stage of *O. sinensis* in the host hemolymph, the transformation stage from blastospore to hypha, and the formation and development stage of fruiting body. According to the report, when *Cordyceps* *militaris* forms fruiting bodies on wheat medium and insects, the stable reference genes are different [7]. We think that the different stage of Chinese cordyceps needs to re-verify the stable of reference genes, so we collect samples at different stages of the formation process of Chinese cordyceps, and also cover the light stress that is often used to improve the phenotype in production. Our study covers almost all stages of the formation of Chinese cordyceps, which can provide a more comprehensive reference for the study of gene expression of *O. sinensis*.

Figure1. Morphology of hyphae. A: Aerial mycelium; B: Hyphal knot; C: Substrate mycelium [6].

1.	Yang J, Lin Q, Lin J, Ye X. Selection and Validation of Reference Genes for Quantitative Real-Time Polymerase Chain Reaction Studies in Mossy Maze Polypore, *Cerrena* *unicolor* (Higher Basidiomycetes). Int J Med Mushrooms. 2016;18: 165–175. doi:10.1615/IntJMedMushrooms.v18.i2.70

2.	Zhou Y-H, Zhang Y-J, Luo Z-B, Fan Y-H, Tang G-R, Liu L-J, et al. Selection of optimal reference genes for expression analysis in the entomopathogenic fungus *Beauveria* *bassiana* during development, under changing nutrient conditions, and after exposure to abiotic stresses. Appl Microbiol Biotechnol. 2012;93: 679–685. doi:10.1007/s00253-011-3561-3

3.	Qian J, Gao Y, Wáng Y, Wu Y, Wāng Y, Zhao Y, et al. Selection and Evaluation of Appropriate Reference Genes for RT-qPCR Normalization of *Volvariella* *volvacea* Gene Expression under Different Conditions. Biomed Res Int. 2018;2018: 6125706. doi:10.1155/2018/6125706

4.	Fu N, Li J, Wang M, Ren L, Zong S, Luo Y. Identification and Validation of Reference Genes for Gene Expression Analysis in Different Development Stages of *Amylostereum* *areolatum*. Front Microbiol. 2022;12: 827241. doi:10.3389/fmicb.2021.827241

5.	Zhao X, Yang H, Chen M, Song X, Yu C, Zhao Y, et al. Reference Gene Selection for Quantitative Real-Time PCR of Mycelia from *Lentinula* *edodes* under High-Temperature Stress. Biomed Res Int. 2018;2018: 1670328. doi:10.1155/2018/1670328

6.	Su Q, Xia Y, Xie F, Vestine U, Chen Z, Zhou G. Screening of the reference genes for qRT-PCR analysis of gene expression in *Ophiocordyceps* *sinensis*. MYCOSYSTEMA. 2021;40: 1712–1722. doi:10.13346/j.mycosystema.210015

7.	Lian T, Yang T, Liu G, Sun J, Dong C. Reliable reference gene selection for *Cordyceps* *militaris* gene expression studies under different developmental stages and media. FEMS Microbiol Lett. 2014;356: 97–104. doi:10.1111/1574-6968.12492

*The language correction is required throughout the manuscript.*

Response: We thank the reviewer for the comments. The manuscript has been re-edited by the language-editing professionals.

**Response to Review Comments**

**Response to Reviewer #1:**

*The present work related to reference gene expression in O. sinensis. Lian et al (2014) ref no 36 also performed the similar work. Here, authors need to perform the detail work in the different light intensity or different light interval (hours or days) for the expression analysis. Beside this manuscript also required proofreading.*

Response: Thank you for your comment. In the study of Lian *et al* (2014), the study species is *Cordyceps militaris* (Ascomycota, Sordariomycetes, Hypocreales, Cordycipitaceae, *Cordyceps*, *Cordyceps* *militaris*), the optimum growth temperature is 25~28 ℃But the species we studied in this study is *O. sinensis* (Ascomycota, Sordariomycetes, Hypocreales, Ophiocordycipitaceae, *Ophiocordyceps*), which is a low-temperature fungus, the optimum growth temperature is 16~20 ℃, when the temperature exceeds 23 ℃, the fungus will autolyse and die [1,2]. There are also great differences in the artificial cultivation and development of the fruiting bodies of the two fungi. For *C. militaris*, fruiting bodies can be induced on wheat medium and pupa, and it takes about 50 days from inoculation of mycelium to fruiting body formation[3]. However, for *O. sinensis*, the fruiting body cannot be stably cultivation in the medium at present, is usually induced by inoculating *Thitarodes xiaojinensis* larvae. It usually takes more than one year from the inoculation of larvae to the formation of fruiting bodies. Therefore, we believe that the stability of reference genes in different development processes of *O. sinensis* needs careful selection and re-evaluation. During the development of *C.* *militaris* fruiting body development, the most stable reference gene is *rpb1*, and our study found that *Try* and *Cox5* are considered to be the best reference genes in *O. sinensis* fruiting body development. Which confirms our conjecture that the development process of *O. sinensis* and *C.* *militaris* is different, and the selection of reference genes needs to be screened again.

Figure 1. Different developmental stages of Cordyceps militaris used for RNA extractions. Five different developmental stages in wheat medium (upper) or pupae (lower) were shown. From left to right: white mycelia under dark (WWM, 7 days after inoculation), orange mycelia with apparent carotenoid accumulation after being exposed to light (WOM, 10 days after inoculation), primordium (WP, 17 days after inoculation), nascent fruit body (WNF, 35 days after inoculation), and fully matured fruit body (WMF, 55 days after inoculation) in the upper and white mycelia under dark (PWM, after inoculation 10 days), sclerotium (PS, 15 days after inoculation), primordium (PP, 22 days after inoculation), nascent fruit body (PNF, 30 day after inoculations), and fully matured fruit body (PMF, 50 days after inoculation) in the lower [3].

Many studies have proved that the expression of common housekeeping genes will change due to different species and experimental treatments, and there were no known stable reference gene applicable to all conditions. Therefore, many researchers have re-selected and verified stable reference genes in corresponding experimental models. In *Cerrena* *unicolor*, the expression levels of seven candidate reference genes were studied at different growth phases, under various induction conditions, and with a range of carbon/nitrogen ratios and carbon and nitrogen sources [4]. Similarly, as early as 2012, stable reference genes of *Beauveria bassiana* at different development stages and under various nutritional and stress conditions have been reported [5]. Subsequently, stable reference genes under different conditions were reported in *Volvariella* *volvacea* [6], *Amylostereum* *areolatum* [7] and *Lentinula* *edodes* [8]. Therefore, for a different species (*O. sinensis* in our study), the stability of reference genes needs to be re-evaluated.

Further, *O. sinensis* is a kind of low-temperature fungus. The optimum temperature for its growth is 16 ℃. The formation and development of fruiting body takes several months (as described in the results in MS), so it is very different from other edible fungi. It would be very imprecise to directly use the reference genes of other fungal development processes or under stress conditions in the study of *O. sinensis*.

So far, there is only one report (the article 1 you quoted above) on the research of reference genes in *O. sinensis* [9]. Their research mainly focused on the stability of reference genes in the asexually proliferated *O. sinensis* in solid medium (Figure 2 below), and the results have good guiding value for the solid fermentation of *O. sinensis*. But we focus more on the formation process of Chinese cordyceps, including the multiplication stage of *O. sinensis* in the host hemolymph, the transformation stage from blastospore to hypha, and the formation and development stage of fruiting body. According to the report, when *Cordyceps* *militaris* forms fruiting bodies on wheat medium and insects, the stable reference genes are different [3]. We believe that the different stage of Chinese cordyceps needs to re-verify the stable of reference genes, so we collect samples at different stages of the formation process of Chinese cordyceps, and also cover the light stress that is often used to improve the phenotype in production. Our study covers almost all stages of the formation of Chinese cordyceps, which can provide a more comprehensive reference for the study of gene expression of *O. sinensis*.

Figure 2. Morphology of hyphae. A: Aerial mycelium; B: Hyphal knot; C: Substrate mycelium [9].

1.	Ge F, Gui L, Li C, Fan M. Studies on solid-state fermentation condition of *Hirsutella* *sinensis* anamorph of *Cordyceps* *sinensis*. .JOURNAL OF BIOLOGY. 2009;26: 22–25. doi:10.3969/j.issn.1008-9632.2009.03.022

2.	Wang S, Zhu X, Zhu P. The Preliminary Cultivation and Observation on the Vitality of *Hirsutella* *sinensis*. EDIBLE FUNGI OF CHINA. 2003;22: 4–6. doi:10.3969/j.issn.1003-8310.2003.06.002

3.	Lian T, Yang T, Liu G, Sun J, Dong C. Reliable reference gene selection for *Cordyceps* *militaris* gene expression studies under different developmental stages and media. FEMS Microbiol Lett. 2014;356: 97–104. doi:10.1111/1574-6968.12492

4.	Yang J, Lin Q, Lin J, Ye X. Selection and Validation of Reference Genes for Quantitative Real-Time Polymerase Chain Reaction Studies in *Mossy* *Maze* *Polypore*, Cerrena unicolor (Higher Basidiomycetes). Int J Med Mushrooms. 2016;18: 165–175. doi:10.1615/IntJMedMushrooms.v18.i2.70

5.	Zhou Y-H, Zhang Y-J, Luo Z-B, Fan Y-H, Tang G-R, Liu L-J, et al. Selection of optimal reference genes for expression analysis in the entomopathogenic fungus *Beauveria* *bassiana* during development, under changing nutrient conditions, and after exposure to abiotic stresses. Appl Microbiol Biotechnol. 2012;93: 679–685. doi:10.1007/s00253-011-3561-3

6.	Qian J, Gao Y, Wáng Y, Wu Y, Wāng Y, Zhao Y, et al. Selection and Evaluation of Appropriate Reference Genes for RT-qPCR Normalization of *Volvariella* *volvacea* Gene Expression under Different Conditions. Biomed Res Int. 2018;2018: 6125706. doi:10.1155/2018/6125706

7.	Fu N, Li J, Wang M, Ren L, Zong S, Luo Y. Identification and Validation of Reference Genes for Gene Expression Analysis in Different Development Stages of *Amylostereum* *areolatum*. Front Microbiol. 2022;12: 827241. doi:10.3389/fmicb.2021.827241

8.	Zhao X, Yang H, Chen M, Song X, Yu C, Zhao Y, et al. Reference Gene Selection for Quantitative Real-Time PCR of Mycelia from *Lentinula* *edodes* under High-Temperature Stress. Biomed Res Int. 2018;2018: 1670328. doi:10.1155/2018/1670328

9.	Su Q, Xia Y, Xie F, Vestine U, Chen Z, Zhou G. Screening of the reference genes for qRT-PCR analysis of gene expression in *Ophiocordyceps* *sinensis*. MYCOSYSTEMA. 2021;40: 1712–1722. doi:10.13346/j.mycosystema.210015

**Response to Reviewer #2:**

*The current study provides an overview of how stable reference genes might be chosen for Ophiocordyceps sinensis under different developmental and light induced conditions. The manuscript is well written and structured. However, the manuscript required several minor corrections before publication.*

*Comments:*

*1. Cite more relevant and recent literature under discussion section.*

Response: Thank you for your comment. In the discussion section, we have We updated some recent references as follows.

| **Old Reference No.** | **Author and year** | **New Reference No.** | **Author and year** |
| --- | --- | --- | --- |
| 28 | (Llanos *et al.* 2015) | 26 | (Qian *et al.* 2018) |
| 30 | (Ren and Yao 2013) | 29 | (Li *et al.* 2020) |
| 31 | (Cao *et al.* 2015) | 12 | (Zhong *et al.* 2018) |
| 18 | (Xiang *et al.* 2014) | 12 | (Zhong *et al.* 2018) |
| 37 | (Jin *et al.* 2010) | 40 | (Luo *et al.* 2019) |

Cao, L., Ye, Y., and Han, R. 2015. Fruiting Body Production of the Medicinal Chinese Caterpillar Mushroom, *Ophiocordyceps* *sinensis* (Ascomycetes), in Artificial Medium. Int J Med Mushrooms **17**: 1107–1112. doi:10.1615/intjmedmushrooms.v17.i11.110.

Jin, K., Zhang, Y., Fang, W., Luo, Z., Zhou, Y., and Pei, Y. 2010. Carboxylate transporter gene JEN1 from the entomopathogenic fungus *Beauveria* *bassiana* is involved in conidiation and virulence. Appl Environ Microbiol **76**: 254–263. doi:10.1128/AEM.00882-09.

Li, M., Meng, Q., Zhang, H., Shu, R., Zhao, Y., Wu, P., Li, X., Zhou, G., Qin, Q., and Zhang, J. 2020. Changes in transcriptomic and metabolomic profiles of morphotypes of *Ophiocordyceps* *sinensis* within the hemocoel of its host larvae, *Thitarodes* *xiaojinensis*. BMC Genomics **21**: 789. doi:10.1186/s12864-020-07209-2.

Llanos, A., François, J.M., and Parrou, J.-L. 2015. Tracking the best reference genes for RT-qPCR data normalization in filamentous fungi. BMC Genomics **16**: 71. doi:10.1186/s12864-015-1224-y.

Luo, Y., Wang, G., Wang, C., Gong, Y., Bian, Y., and Zhou, Y. 2019. Selection and Validation of Reference Genes for qRT-PCR in *Lentinula* *edodes* under Different Experimental Conditions. Genes (Basel) **10**: 647. doi:10.3390/genes10090647.

Qian, J., Gao, Y., Wáng, Y., Wu, Y., Wāng, Y., Zhao, Y., Chen, H., Bao, D., Xu, J., and Bian, X. 2018. Selection and Evaluation of Appropriate Reference Genes for RT-qPCR Normalization of *Volvariella* *volvacea* Gene Expression under Different Conditions. Biomed Res Int **2018**: 6125706. doi:10.1155/2018/6125706.

Ren, S.-Y., and Yao, Y.-J. 2013. Evaluation of nutritional and physical stress conditions during vegetative growth on conidial production and germination in *Ophiocordyceps* *sinensis*. FEMS Microbiol Lett **346**: 29–35. doi:10.1111/1574-6968.12190.

Xiang, L., Li, Y., Zhu, Y., Luo, H., Li, C., Xu, X., Sun, C., Song, J., Shi, L., He, L., Sun, W., and Chen, S. 2014. Transcriptome analysis of the *Ophiocordyceps* *sinensis* fruiting body reveals putative genes involved in fruiting body development and cordycepin biosynthesis. Genomics **103**: 154–159. doi:10.1016/j.ygeno.2014.01.002.

Zhong, X., Gu, L., Wang, H., Lian, D., Zheng, Y., Zhou, S., Zhou, W., Gu, J., Zhang, G., and Liu, X. 2018. Profile of *Ophiocordyceps* *sinensis* transcriptome and differentially expressed genes in three different mycelia, sclerotium and fruiting body developmental stages. Fungal Biol **122**: 943–951. doi:10.1016/j.funbio.2018.05.011.

In addition, we also revised the discussion section of MS and added the following references.

Li M, Meng Q, Zhang H, Shu R, Zhao Y, Wu P, et al. Changes in transcriptomic and metabolomic profiles of morphotypes of *Ophiocordyceps* *sinensis* within the hemocoel of its host larvae, *Thitarodes* *xiaojinensis*. BMC Genomics. 2020;21: 789. doi:10.1186/s12864-020-07209-2

Lian T, Yang T, Liu G, Sun J, Dong C. Reliable reference gene selection for *Cordyceps* *militaris* gene expression studies under different developmental stages and media. FEMS Microbiology Letters. 2014;356: 97–104. doi:10.1111/1574-6968.12492

Lyu X, Jiang S, Wang L, Chou T, Wang Q, Meng L, et al. The Fvclp1 gene regulates mycelial growth and fruiting body development in edible mushroom *Flammulina* *velutipes*. Arch Microbiol. 2021;203: 5373–5380. doi:10.1007/s00203-021-02514-0

Rodenburg SYA, Terhem RB, Veloso J, Stassen JHM, van Kan JAL. Functional Analysis of Mating Type Genes and Transcriptome Analysis during Fruiting Body Development of *Botrytis* *cinerea*. mBio. 2018;9: e01939-17. doi:10.1128/mBio.01939-17

Zhang C, Shang D, Zhang Y, Gao X, Liu D, Gao Y, et al. Two Hybrid Histidine Kinases Involved in the Ethylene Regulation of the Mycelial Growth and Postharvest Fruiting Body Maturation and Senescence of *Agaricus* *bisporus*. Microbiol Spectr. 2022;10: e02411-22. doi:10.1128/spectrum.02411-22

*2. Reference no. 4, 13, 21, 24, 31, 32, 33: correct the references.*

Response: Thank you for your comment. We have replaced the references with the latest published.

*3. Line no 171, 341: write the scientific name in italics.*

Response: Thank you for your comment. We have revised the words according to the review comments. The scientific name in line 171 has been corrected to italic in line 201. The scientific name in line 341 is in reference [7] (Tao *et al.*, 2016), and this reference has been replaced with reference [6] (Liu *et al.*, 2019) in new MS.

*4. Line no. 218-219: avoid the repetitions.*

Response: Thank you for your comment. We have deleted and revised the repeated sentences in line 247-248.

*5. Line no 382: write the name of genes in italics.*

Response: Thank you for your comment. We have revised the name of genes in italics in line 527.

*6. Table no 2, 3: check the alignment.*

Response: Thank you for your comment. We have revised the Tables.

**Response to Reviewer #3:**

*Required grammer check or language editing must improved. Imporve the quality of paper by following the comments attached in this link. Please ensure that you follow PLOS one guidelines for presenting name of authors/abbreviation of journals/year of publication in bold/volume journals in italics etc., in the refeerence section. and also in other sections.*

Response: Thank you for your comment. According to your suggestion, we have revised the syntax error and description of MS. And the manuscript has been re-edited by the language-editing professionals. According to the requirements of PLOS one guidelines, we have revised the format of references.

*Line 21~24: Which gene expression? Please Include. Include the purpose of selection of those expression stability.*

Response: Thank you for your comment. We rewrote the relevant part of the abstract, and listed the names of the reference genes and the purpose of verifying their stability, in line 19-28 as follows.

*The formation process of Chinese cordyceps includes two parts: asexual proliferation (Ophiocordyceps sinensis proliferates in the hemolymph of Thitarodes armoricanus larvae) and sexual development (formation and development of fruiting bodies). Therefore, validation of reference genes under different development stages and experimental conditions is crucial for RT-qPCR analysis. However, there is no report on stable reference genes at the development stage of O. sinensis fruiting body. In this study, ten candidate reference genes, Actin, Cox5, Tef1, Ubi, 18s, Gpd, Rpb1, Try, Tub1 and Tub2, were selected and calculated their expression stability using four methods: geNorm, NormFinder, BestKeeper, and Comparative △Ct.*

*Line 28~31: Please correct the sentences.*

Response: Thank you for your comment. We have revised the sentences as follows in line 32-35.

*Our study provides a guidance for reference genes selections at different proliferation processes with light stress of O. sinensis, and represents a foundation for studying the molecular mechanism of Chinese cordyceps formation.*

*Line 35: Check it*

Response: Thank you for your comment. We revised ”Dong Chong Xia Cao, ” to “Dong Chong Xia Cao” in line 39.

*Line 51: What development include it.*

Response: Thank you for your comment. We revised *development* to *fruiting body development* in line 53-54.

*Line 57~58: Correct the sentences. Include the purpose of importance to study.*

Response: Thank you for your comment. We have revised the sentences as follows in line 60-62.

*A large amount of omics data provided many new targets for O. sinensis artificial cultivation. However, no stable reference gene has been reported, which is very important for studying the molecular mechanism.*

*Line 58~60: Include the purpose of importance to study.*

Response: Thank you for your comment. We added the purpose of importance to study as follows in line 63-67.

*Studying the key genes and molecular mechanisms in the process of O. sinensis infection, asexual proliferation, sexual development, and synthesis of active substances will become the research focus of artificial cultivation of Chinese cordyceps. During the formation of Chinese cordyceps, we usually focus on the formation of fruiting bodies and the effect of light on O. sinensis.*

*Line 104: Don’t use these of type of words., Please correct it. What is the purpose of these selection on what basis?*

Response: Thank you for your comment. We have revised the sentences as follows in line 122-123, explained the reasons for selecting genes and cited relevant references.

*According to similar studies in other fungi [18,19,25,26], ten housekeeping genes were selected as candidate reference gene targets.*

*Line 128: Include how the expression pattern was analyzed. What representation?*

Response: Thank you for your comment. We revised the relevant statements, explained how to group in the RT-qPCR section (line 137-140) as follows,

*The samples were divided into three groups: asexual proliferation (all liquid fermentation samples), sexual proliferation (samples during fruiting body development), and light treatment (light- and dark-treated samples during liquid fermentation and fruiting body development).*

and how to analyze the expression pattern in the Statistical analysis section (line 148-163) as follows.

*The expression stability of ten candidate reference genes was analyzed using geNorm, NormFinder, BestKeeper, and Comparative △Ct methods. GeNorm program converts cycle number (Ct) into relative gene expression (2ΔCt), evaluated by calculating the stable value (M) of candidate reference gene expression, and finally, two or more reference gene combinations were provided by the analysis results of pairwise variation (Vn/Vn+1) [21]. NormFinder is also sorts the stability of reference genes according to M, and only one stable reference gene can be provided [22]. BestKeeper evaluated the stability of reference gene expression according to standard deviation (SD) and coefficient of variation (CV), the more stable genes have lower SD values [23].△Ct method is to compare the △Ct values of paired genes in different samples. If the △Ct values of these two genes remain unchanged in all samples, it is considered that these two genes are stably expressed in these samples; Otherwise, the expression of one or two genes is considered unstable. The final gene stability is sorted according to the change of △Ct value [24]. The overall final ranking was calculated by RefFinder(http://blooge.cn/RefFinder/) [27], which assigns the appropriate weights to individual genes based on the ranking of each program and calculates the geometric mean of their weights for the overall final ranking.*

*Line 145~147: Improve the sentence. Whether the intended sentence is retained or not.*

Response: Thank you for your comment. We have revised the sentences as follows in line 173-175.

*The mycelial pellets become dense and smooth after 70 days of culture, and appear white (Fig 1A-d) and black (Fig 1A-e) respectively under the induction of dark and light conditions.*

*Line 151~152: Why it not change? What is the reason behind it? Include it.*

Response: Thank you for your comment. At this stage, the *O. sinensis* proliferation in the host, and the mycelium does not penetrate the host epidermis, so the appearance of the mummified larvae does not change much. We have revised the sentences as follows in line 179-181.

*Initially, O. sinensis only proliferates in the host, and the mycelium does not penetrate the host epidermis so that the head shell of the mummified larvae (Fig 1B-b) did not change significantly.*

*Line 168: There is not figure in this manuscript. Please add it. If added kindly ignore?*

Response: Thank you for your comment. We uploaded high-quality pictures in the manuscript submission system. We checked the final PDF file building from the submission system and confirmed that it contains all the figures of MS.

*Line 187: Please alternate the sentence or correct it.*

Response: Thank you for your comment. We have revised the sentences as follows in line 217-219.

*The samples were divided into three groups (asexual proliferation, sexual proliferation, and light treatment), and the candidate genes had the same expression trend in different groups.*

*Line 253: Generally, this section needs to be supplemented with more other literature review papers. I think you should discuss the innovations and more clarifying why your study is important, explain in more details.*

Response: Thank you for your comment. We have revised the discussion section, supplemented the literature of similar species in fungi. And the importance and innovation of this study are discussed by comparing different studies, in line 335-346.
